# Immune checkpoint inhibition for pancreatic ductal adenocarcinoma: limitations and prospects: a systematic review

**DOI:** 10.1186/s12964-021-00789-w

**Published:** 2021-11-24

**Authors:** Hong-Bo Li, Zi-Han Yang, Qing-Qu Guo

**Affiliations:** Department of Gastrointestinal Surgery, The Second Affiliated Hospital of Zhejiang University School of Medicine, 88 Jiefang Road, Hangzhou, 310009 Zhejiang Province China

**Keywords:** Pancreatic ductal adenocarcinoma, Immune checkpoint inhibitors, Tumor microenvironment

## Abstract

Pancreatic cancer is an extremely malignant tumor with the lowest 5-year survival rate among all tumors. Pancreatic ductal adenocarcinoma (PDAC), as the most common pathological subtype of pancreatic cancer, usually has poor therapeutic results. Immune checkpoint inhibitors (ICIs) can relieve failure of the tumor-killing effect of immune effector cells caused by immune checkpoints. Therefore, they have been used as a novel treatment for many solid tumors. However, PDAC is not sensitive to monotherapy with ICIs, which might be related to the inhibitory immune microenvironment of pancreatic cancer. Therefore, the way to improve the microenvironment has raised a heated discussion in recent years. Here, we elaborate on the relationship between different immune cellular components in this environment, list some current preclinical or clinical attempts to enhance the efficacy of ICIs by targeting the inhibitory tumor microenvironment of PDAC or in combination with other therapies. Such information offers a better understanding of the sophisticated tumor-microenvironment interactions, also providing insights on therapeutic guidance of PDAC targeting.

**Video Abstract**

**Supplementary Information:**

The online version contains supplementary material available at 10.1186/s12964-021-00789-w.

## Background

As early as 2014, some researchers predicted that by 2030 pancreatic cancer would surpass breast and colorectal cancer as the second-largest tumor-related fatal disease [1]. PDAC is the most common histological subtype [2] and has a mortality rate almost equal to its incidence rate. In 2019, 45,750 of the 56,770 newly diagnosed pancreatic cancer patients in the USA will eventually die from the disease (American Cancer Society. Cancer Facts and Figures 2019; American Cancer Society: New York, NY, USA, 2019). There are three main reasons for poor prognosis of PDAC: (1) lack of specific tumor markers and early screening methods leads to late diagnosis; (2) distant metastasis occurs early, and patients often lose the opportunity for surgery; and (3) pancreatic cancer, as a “cold” tumor, has a poor response to radiotherapy and chemotherapy [3]. Surgical resection combined with chemoradiotherapy may prolong the overall survival (OS) time of patients with localized disease; however, its effect on patients with advanced-stage is still unsatisfactory [4]. Hence, considering the fact that existing treatment cannot completely cure pancreatic cancer, patients urgently need a more effective treatment. ICIs can block the co-inhibitory signaling pathway in tumor cells and promote immune-mediated tumor cell clearance [5]. ICIs have also been proved to be effective against a variety of solid tumors, including melanoma [6–8], as well as lung [9–11], renal [12, 13] and bladder [14, 15] cancer. However, this new treatment seems not to be entirely effective for pancreatic cancer, at least with monotherapy [16], which might be related to the unique immunosuppressive tumor microenvironment (TME) of PDAC. Therefore, reversing the silent TME to make tumors sensitive to ICI therapy may be a new effective treatment for PDAC. Therefore, in order to better apply ICI drugs to the clinical treatment of PDAC patients, we try to describe the research progress of ICI drugs in clinical and laboratory, and some assumptions of reversing the immunosuppressive pancreatic TME by targeting immune cells and small molecules, and provide some future directions to improve the therapeutic efficacy for later researchers.

## The mainly immunosuppressive cells in the TME of PDAC

### Regulatory T cells (Tregs)

Tregs were first identified by Sakaguchi [17] and are indispensable for the maintenance of normal immune tolerance, and their deficiency leads to many autoimmune diseases [18]. However, Tregs in the TME often aggravate immunosuppression and hinder immunotherapy [19]. Tregs exert their immunosuppressive effect through two completely different pathways, tumor-intrinsic and tumor-extrinsic pathways [19]. The internal regulatory function of Tregs mainly depends on the secretion of immunosuppressive cytokines, such as transforming growth factor-β (TGF-β), interleukin (IL)-10, IL-35, and depleted IL-2, which downregulates proliferation of effector T cells. Thus, the killing effect of effector T cells on tumor cells is reduced [20–26]. For the extrinsic pathway, immune checkpoint molecules, such as cytotoxic T lymphocyte-associated protein (CTLA-4), are expressed on the membrane of Tregs [27]. These molecules have a high affinity for CD80/CD86 on effector cells [19]. The binding of these two receptors can lead to the following results. Firstly, competitive inhibition of the binding of the CD28 receptor to B7 on the surface of traditional T cells can inhibit the activation of T cells [19]. Secondly, indoleamine 2,3-dioxygenase (IDO) can be induced by Tregs, the rate-limiting enzyme for tryptophan metabolism to kynurenine, and further leads to the apoptosis of effector T cells caused by tryptophan deficiency [28]. Other evidence also shows that Treg affects the function of effector T cells. For example, in the tumor model of Treg removed mice, the immunosuppression of tumor-infiltrating CD8^+^ cells was relieved [29]. Finally, the binding of CTLA-4 and B7 also downregulates the number of B7 receptors on the surface of dendritic cells (DCs), which further hinders the inhibitory effect of functional T cells on the immune response [30]. In addition to CTLA-4, Tregs in the TME overexpress many other immunosuppressive molecules, including glucocorticoid-induced TNFR-related protein (also known as TNFRSF18), lymphocyte-activation gene (LAG)3 protein, T cell immunoglobulin mucin receptor 3 (TIM, also known as HAVCR2), OX40 (also known as TNFRSF4), programmed cell death protein (PD)-1, and inducible T-cell co-stimulator (ICOS) [31–33]. The complex interaction between these molecules and other components in the TME makes Tregs become a barrier in the process of immune recognition and elimination of tumor cells by effector cells (Additional file 4).

### Myeloid-derived suppressor cells (MDSCs)

In the tumor, trauma, and other pathological states, bone marrow progenitor cells and immature myeloid cells cannot differentiate into normal mature granulocytes but form a kind of immature heterogeneous cells [34], namely MDSCs. MDSCs can be divided into two subtypes, polymorphonuclear (PMN)-MDSCs and monocytic-MDSC (M-MDSC). These two subtypes have immunosuppressive effects, and M-MDSCs have stronger immunosuppressive ability [35]. Once these immunosuppressive cells are recruited into the TME, they can be activated by the surrounding vascular endothelial growth factor (VEGF). Furthermore, activated MDSCs can produce more VEGF, which is a positive feedback process [36]. Activated MDSCs mediate immunosuppression in the TME, mainly through the consumption of amino acids necessary for the proliferation of immune cells, as well as the release of reactive oxidants such as induced NO synthase and NAPDH oxidase 2, and ultimately affect the activity of effector T cells [37, 38]. An experiment conducted by Stromnes et al. [39] showed that depletion of granulocytic MDSC (GR MDSC) in PDA models in vivo and in vitro could increase the internal accumulation of activated CD8^+^ T cells and apoptosis of tumor epithelial cells.In addition, MDSCs can induce Treg proliferation by secreting IL-2 and TGF-β to mediate immunosuppression indirectly [40]. MDSCs can also upregulate the expression of programmed death-ligand (PD-L)1 [41] and promote the proliferation of Tregs, which is regulated by IL-10 secreted by activated T cells in the TME [40].

### Tumor-associated macrophages (TAMs)

TAMs, one of the immune cell populations abundant in TME, are referred to the macrophages in the tumor stroma, which can be divided into two types, the M1 and M2, according to their phenotypes and functions in the view of macrophage polarization [42]. In general terms, M1 macrophages are pro-inflammatory with antitumor properties, while M2 macrophages are anti-inflammatory with both antitumor and protumor properties in TME [43]. However, the M2 type deviation with protumor effects is predominant in PDAC. Monocytes entering the TME under the influence of chemotaxis differentiate into TAMs [44]. TAMs exert their immunosuppressive function mainly through the expression of ligands or receptors. Like MDSCs, TAMs also express arginase-1 and IDO, leading to depletion of essential amino acids for T cell proliferation. In addition, TAMs overexpress PD-1, PD-L1, and HLA, which are the ligands of inhibitory receptors such as CD94 and IL-2/4. The binding of PD-1 to PD-L1 contributes to immune escape and T-cell depletion, while the binding of HLA to the inhibitory receptor IL-2/4 on the surface of T cells can directly inhibit the proliferation of T cells [45, 46]. IL-10, TGF-1, and prostaglandin E2 in the TME can inhibit the expression of MHC-II molecules on the surface of macrophages, so the macrophages cannot effectively present tumor antigen to T cells and hinder the specific killing reaction of T cells against tumor cells [46, 47]. In addition, Beavis et al*.* found that TAMs can overexpress CD73 and CD39 ectoenzymes and generate pericellular adenosine, which finally causes the suppression of Teff via activation of the adenosine A2A receptor [48], suggesting that TAMs play an important role in the immunosuppressive pancreatic TME.

### Pancreatic satellite cells (PSCs)

The most significant difference between pancreatic cancer and other solid tumors is that there are 80%–90% matrix components in pancreatic cancer tissue [47]. In health, PSCs are responsible for maintaining the homeostasis of the extracellular matrix proteins. Recent studies have implied its potential immune functions in normal. Apte et al*.* found that quiescent PSCs can phagocytize damaged pancreatic parenchymal cells, and this can delay the progression of early pancreatic disease. However, a variety of small molecules, including cytokines such as hyperglycemia, endothelin 1, COX-2, galectin 1, and fibrinogen, can activate PSCs through the paracrine pathway [49]. Activated PSCs secrete many matrix proteins containing type I collagen, which plays a crucial role in the formation of the dense extracellular matrix of pancreatic cancer. The dense extracellular matrix constitutes a physical barrier in the TME of pancreatic cancer, blocks the infiltration of T and B lymphocytes, and affects the recognition and elimination of tumor cell antigens by these lymphocytes. In addition to participating in the formation of pancreatic cancer inhibitory TME, PSCs can also secrete various inhibitory cytokines to participate in the regulation of immune cells. For example, granulocyte–macrophage colony-stimulating factor (GM-CSF) and IL-6 secreted by PSCs can directly inhibit the migration and invasion of cytotoxic T cells and reduce the number of cytotoxic T cells in the TME. In contrast, IL-6 can induce MDSCs into the TME and exert an immunosuppressive effect [50, 51]. Galectin-1, another cytokine highly expressed in PSCs, plays an essential role in maintaining the immunosuppressive TME and T-cell depletion [52]. PSCs significantly increase the number of other immunosuppressive cells in the PDAC microenvironment, including MDSCs, M2-TAMs, and Tregs, and decrease the number of effector T cells, natural killer (NK) cells, and M1 TAMs. Also, Ene-Obong et al. [53] reported that in KPC (Pdx-1-Cre; LSL-KrasG12D/ + ; LSL-Trp53R172H/ +) mice, PSCs sequester antitumor CD8^+^ T cells around nonadjacent regions in the stroma, resulting in the dysfunction of CD8^+^ cells to infiltrate into the pancreatic tumor epithelial cells. Therefore, PSCs aggravate the immunosuppression of PDAC [54]. However, another study showed that depletion of carcinoma associated fibroblasts would induce the process of immunosuppression and accelerates pancreas cancer, suggesting that PSC may have antitumor properties at the same time [55]. Thus, the exact role of PSC in Pancreatic TME remains ambiguous. (The mechanism of endothelial cell entry into the pancreatic cancer microenvironment and its immunosuppressive effect is summarized in Additional file 1.)

### Interacting between different compounds of PDAC microenvironment

**Fig. 1:**
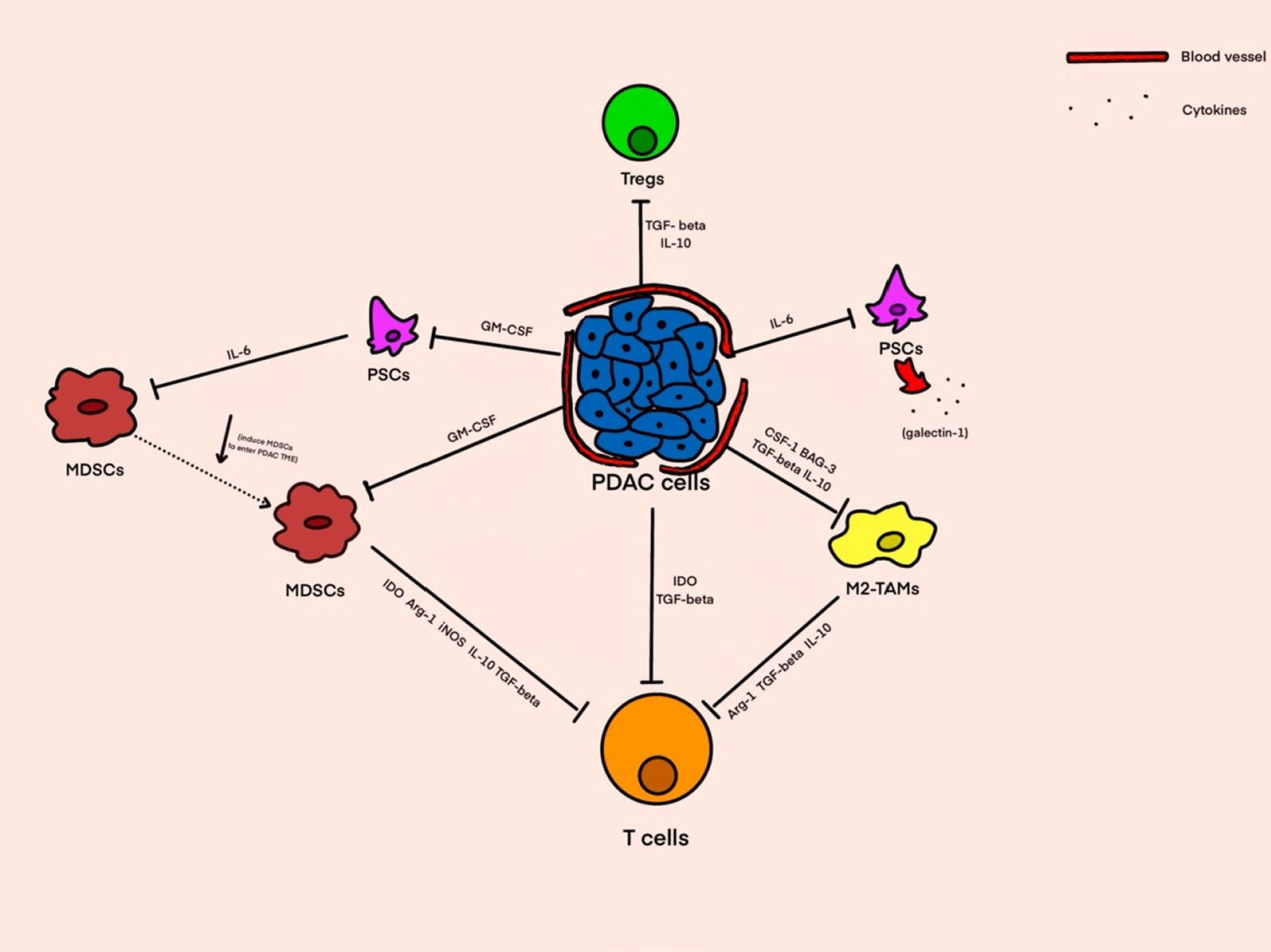
The relationship between different cellular components in the tumor microenvironment of PDAC

As the core of the tumor microenvironment in pancreatic cancer, PDAC cells can directly or indirectly inhibit the immune function of T cells [56], and the direct effects include the secretion of inhibitory cytokines such as IDO and TGF-β, which directly inhibit the proliferation of T cells. The indirect inhibitory effect is related to the interaction of various immunosuppressive cell components in the tumor microenvironment of PDAC, PDAC cells can promote the proliferation and activation of MDSC by secreting GM-CSF, and MDSC can continue to secrete cytokines such as IDO, IL-10, TGF-β, Arg-1, iNOS to inhibit the cell activity and immune effect of T cells. TAMs can also receive cytokines such as CSF-1, Bag-3, TGF-β, and IL-10 secreted by PDAC and then secrete inhibitory cytokines such as Arg-1, TGF-β, and IL-10 similar to MDSCs, resulting in T cell immune dysfunction. As the representative cells negatively regulate the body’s immune function, Tregs are also regulated by TGF-β and IL-10 secreted by PDAC cells. All of these reflect the core role of PDAC cells in the immunosuppressive tumor microenvironment of pancreatic cancer. PSC cells not only promote the accumulation of extracellular matrix and participate in the interstitial components of PDAC, but also secrete IL-6 and GM-CSF to promote the proliferation of PDAC cells. At the same time, IL-6 can also induce MDSC cells to enter the tumor microenvironment and activate [57] (Fig. 1).

## Research progress on IC molecules

### CTLA-4 and PD-1

CTLA-4 and PD-1, as two well-known IC molecules, have completely different mechanisms for T cell immune regulation. Both CTLA-4 and CD28 are expressed on the surface of Tregs [58]. CD28 plays an active role in the activation of T cells by binding to B7 ligands (B7-1/2) on the surface of antigen-presenting cells (APCs) [59]. CTLA-4, as the first discovered IC molecule [60], can also bind to B-7 ligands with higher affinity. Thus, CTLA-4 can competitively inhibit the binding of the CD28 receptor to B-7 ligands and further block the vital signal transmission of T-cell activation, leading to an immunosuppressive effect [61, 62]. However, unlike CTLA-4, PD-1 is widely expressed on the surface of activated lymphocytes (B or T cells), NK cells, and many other immune cells [63]. As a member of the CD28 superfamily [64], PD-1 directly inhibits T-cell activation by binding to its two ligands (PD-L1 and PD-L2) [65, 66], which are widely expressed on the surface of tumor cells and many immune cells [67]. The combination between PD-1 and its ligands can induce apoptosis of lymphocytes and finally cause the immune escape of the tumor [64]. Once PD-1 binds to PD-L1, this signal will generate a positive feedback loop to inhibit T cell activation by recruiting SHP2 tyrosine phosphatase. At the same time, this pathway dephosphorylates CD28 and weakens the TCR signal [68]. Other studies have shown that even as two independent immunosuppressive molecules, PD-1 and PD-L1 can independently inhibit the activity of T lymphocytes and reduce the ability of these tumor killer cells to infiltrate the TME [69, 70].

As a critical negative regulator, CTLA-4 limits immune responses of T cells to PDAC cells under the circumstances of pancreatic cancer, which provides a potential treatment option, that is, the CTLA-4 blockade. It is widely acknowledged that by using anti-CTLA-4 antibodies, ipilimumab for instance, the silent immune responses will be restored. The immune system comes back online, followed by tumor regression. In a similar way but with distinct mechanisms of action, by targeting PD-1 using anti-PD-1 antibodies such as nivolumab, tumor regression can also be achieved in cancer patients [71]. In addition, many studies have shown that the high expression of immune checkpoints on the surface of endothelial cells is related to the poor prognosis of PDAC patients, Cloutier et al*.* confirmed by immunohistochemistry that upgrade the expression of PD-L1 will lead to inferior prognosis (*P* = 0.0367), Gao Jin et al*.* revealed that the expression of PD-L1 was related to the T stage of PDAC. The research showed that the positive rate of PD-L1 in patients with PDAC in the T3-T4 stage was much higher than that in patients in the T1-T2 stage [72–74]. Therefore, targeting such IC molecules as immunotherapy strategies provides insights on the immune regulation of TME in PDAC.

### LAG-3 (CD223)

MHC receptors on the surface of APCs bind to T-cell receptors (TCRs) and play an active role in the activation and proliferation of T cells. LAG-3 has a high affinity for MHC class II, which prevents the same MHC molecule from contacting TCRs, thus indirectly hindering TCR signal transduction immune response [75]. LAG-3 is expressed on CD4^+^ T cells, CD8^+^ T cells, Tregs, NK cells, and B cells. The wide expression of LAG-3 not only reduces the activity of CD4^+^ T cells, but also weakens the ability of cytotoxic T cells to eliminate tumor cells. Moreover, LAG-3 can promote the immunosuppressive activity of Tregs through the secretion of TGF-β, IL-10, and other immunosuppressive molecules [76].

### TIM-3

TIM-3 is a member of the Tim gene family [77]. Pu-Ji et al*.* found that the expression of TIM-3 was significantly higher in pancreatic cancer than in healthy pancreas tissue, according to the result of immunohistochemical analysis of patient samples. Similar to PD-1, Tim-3 exerts its immunosuppressive effect by binding with the ligands on the effective immune cells and in a variety of solid tumors, including pancreatic cancer, TIM-3, and PD-1 co-expressed on TILs, resulting in poor clinical prognosis [78]. Its ligands include protein ligands such as galectin-9 [79], carcinoembryonic antigen cell adhesion molecule 1 [80], high mobility group box 1 [81] and non-protein ligand phosphatidylserine [82]. Interferon (IFN)-γ can promote NK cell activity and enhance antigen presentation in favor of the recognition and killing of tumor cells by lymphocytes. TIM-3 is expressed chiefly on IFN-γ-producing CD4^+^ T cells (T helper 1 cells) [83]. By binding with various ligands, TIM-3 can induce CD4^+^ T-cell depletion and reduction of IFN-γ, indirectly inhibiting the activation of immune cells [84].

### T cell immunoglobulin and ITIM domain (TIGIT)

Yu et al*.* [85] first discovered that TIGIT could inhibit T-cell activation as an IC in 2009. TIGIT directly inhibits T-cell activation by directly combining with its ligands CD155 and CD112to transmit inhibitory signals. At the same time, TIGIT can competitively inhibit the binding of CD266 or CD96 with CD155 and CD112, reducing the active signal to T cells [86–88]. The binding of TIGIT with CD155, in turn, induces the phosphorylation of CD155 and release of IL-10, which prevents T cells’ activation [84].

### V-domain Ig-containing suppressor of T-cell activation (VISTA)

VISTA, which is a member of the B7 family, is homologous with PD-L1 [89]. VISTA is highly expressed in PDAC and in endothelial cells and immune cells such as T cells [90]. Expression of VISTA on T cells can inhibit the proliferation and activation of T cells. In addition, Jorge et al*.* in 2019 found that VISTA is highly expressed in CD68^+^ macrophages of PDAC and plays an important role in the reduction of cytokine production by T cells in metastatic pancreatic tumors [91], and Blando et al. [92] found that VISTA was highly expressed in the pancreatic stromal area and diminishes cytokine production by T cells.

### B7-H3

In 2001, Chapoval et al. [93] first found that B7-H3 (also called CD276) can play a positive role in promoting T-cell activation and IFN-γ secretion. However, later studies showed that B7-H3, as a member of the B7 family, acts more as a negative regulator to inhibit the immune response of T cells [94, 95]. B7-H3 is widely expressed on the surface of a variety of activated immune cells, including T cells, NK cells, and APCs [88]. Although no receptor of B7-H3 has been found, its effect on inhibiting T cells and NK cells has been confirmed [96].

### BTLA (CD272)

As a member of the CD28 superfamily [97], BTLA (CD272) is expressed on the surface of T cells, B cells, and MDSCs [98]. BTLA can compete with two TNF family members, LIGHT and lymphotoxin-α (CD160), to bind their ligand, herpesvirus entry mediator (HVEM). CD160, like BTLA, inhibits the activation of T cells after binding with HVEM, while LIGHT promotes the activation of T cells [88]. The combination of BTLA with HVEM inhibits the activation and proliferation of CD4^+^/CD8^+^ cancer-specific T cells by promoting the phosphorylation of immunoreceptor tyrosine-based inhibition motifs (ITIMs) and Srchomology 2 (SH2) domain-containing phosphatase 1 (SHP-1)/SHP-2 association [99].

### Peripheral TCR profiling correlated with responses of ICIs

The TCR is a polymorphic receptor that is essential for the development and the peripheral maturation and activation of T cells. CTLA-4, as a TCR expressed on CD4^+^ and CD8^+^ T cells, competitively inhibits the CD28 co-stimulation, thus inhibiting T cell activation, while PD-1 acts in a distinct manner by preventing CD8^+^

**Fig. 2:**
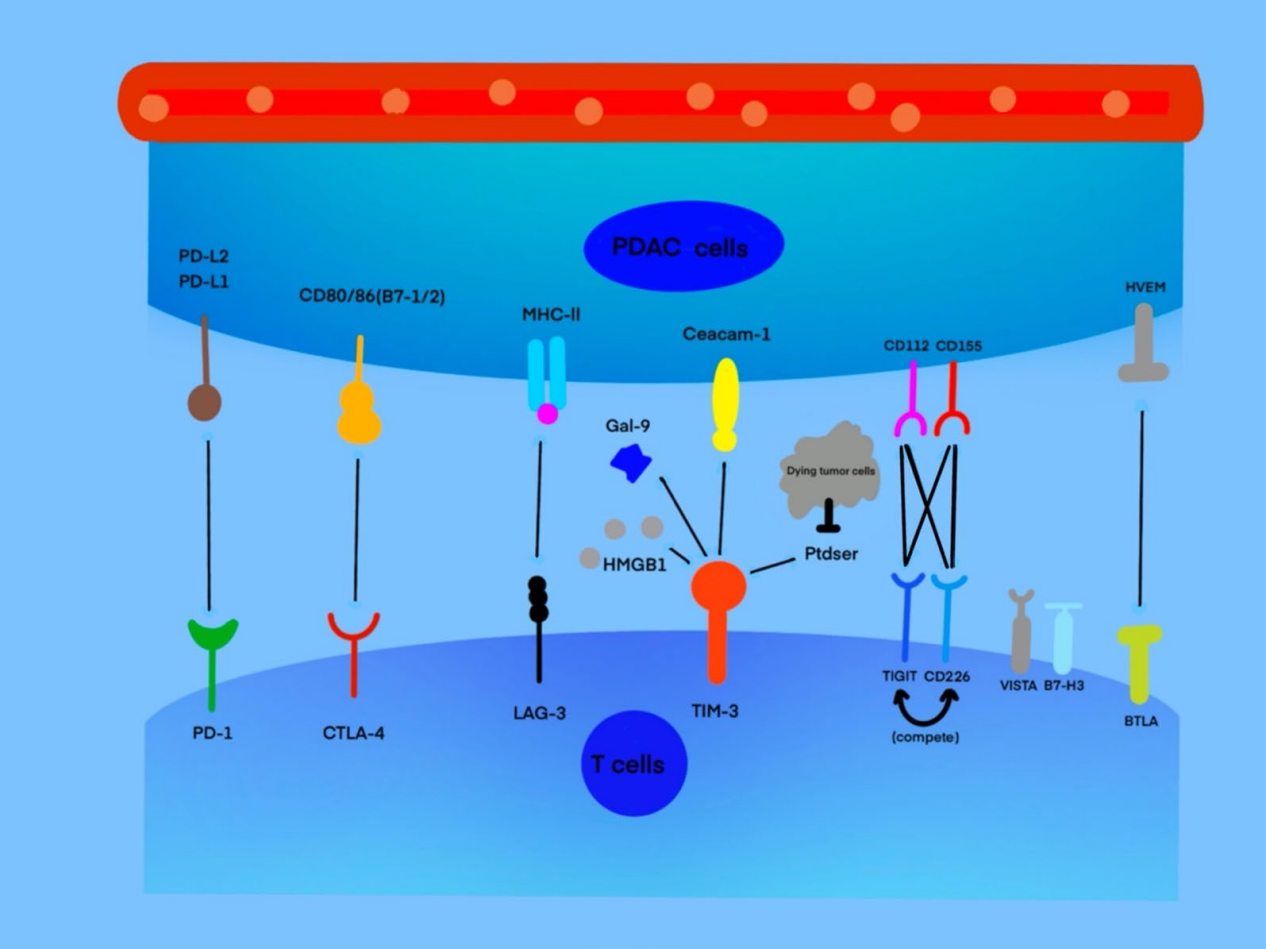
The pairing relationship between various immune checkpoints and their corresponding receptors

T cells from interacting with the target cell. Inhibition of the above two pathways restores the ability of T cells, having them engage and destroy the targets. The development of ICIs to tackle the immune suppression problem improves the efficacy of cancer treatment. Additionally, it brings us a whole new angle to view and assess how the use of ICIs would lead to alterations of the peripheral TCR diversity. Advancements in TCR sequencing and the use of bioinformatic tools allow us to measure the heterogeneity of the T cells, or TCR repertoires [100]. In PDAC patients, a previous study had measured large shifts in TCR repertoire when ICIs involved, which has also been used as predictors of clinical outcome [101]. For instance, anti-CTLA-4 antibodies and anti-PD-1 antibodies both achieve an optimal therapeutic effect in PDAC patients, but each method has different effects on the peripheral TCR repertoire, more specifically, demonstrating a diversification indicated by a change in clonality [101]. Therefore, the evaluation of peripheral TCR repertoire would be a promising direction to elaborate the response of ICIs and further elucidate the rationales of other potential treatments (Fig. 2).

## Research progress on treatment with ICIS

### Research progress of ICI monotherapy

Ipilimumab, an inhibitor of CTLA-4, can improve the prognosis of patients with malignant melanoma and is approved by the USA and Europe for clinical use [6, 102]. Research on ipilimumab for pancreatic cancer has also been underway for several years. In 2010, Richard et al*.* used ipilimumab as monotherapy for locally advanced or metastatic PDAC. However, of the 27 patients who participated in the experiment, only two with locally advanced disease showed mild efficacy, and the rest of the patients progressed rapidly and died soon thereafter [16]. In a phase II clinical trial (NCT02527434) started in 2015, another CTLA-4 inhibitor, tremelimumab, was used as monotherapy for PDAC. Unfortunately, the average OS and average progression-free survival (PFS) of 20 patients in this clinical trial were 4.14 and 1.84 mo, respectively, which was lower than the patients treated with chemotherapy (8.3 and 4.3 mo). In 2012, Julie et al*.* conducted a clinical trial of PD-L1 antibody monotherapy for a variety of advanced solid tumors, among which 14 patients with PDAC showed no objective responses [103]. After that, two phase I/II clinical trials using anti-PD-1 and anti-PD-L1 as monotherapy for PDAC did not achieve satisfactory results [104, 105]. A phase II clinical trial utilizing another antibody against PD-1, nivolumab, for treatment of metastatic pancreatic cancer is still in progress. In addition to CTLA-4, PD-1, and PD-L1 antibodies, some new IC antibodies have also been used in clinical trials for the treatment of PDAC. However, recent studies have shown that monotherapy with novel ICIs does not significantly improve the condition of cancer patients either. There are two main reasons that lead to the poor efficacy of ICI monotherapy. The first one is that the immunosuppressive pancreatic cancer microenvironment and dense stromal impede the infiltration of effector T cells. Second, the Subtype as MSI-high (MSI-H) or mismatch-repair-deficient (dMMR), which has been confirmed to be effective for ICI drugs, seems quite rare in pancreatic cancer [106]. Although ICI monotherapy for solid tumors, including PDAC, is not effective, these studies have revealed the unique natural immunosuppressive TME of pancreatic cancer. Therefore, how to improve the efficacy of ICI drugs in PDAC, which is called an immune desert by scholars, has become a hot spot in recent years.

### Targeting different components of the TME may enhance the efficacy of ICIs

Failure of monotherapy makes people realize that the use of a single ICI cannot change the immunosuppressive TME of PDAC [107]. Therefore, researchers expect to reverse the inhibitory TME and increase the efficacy of ICIs by targeting different components of the TME, including MDSCs, TAMs, Tregs, and PSCs. MDSCs have been widely studied in recent years as important components of the immunosuppressive TME. Inhibition of C-X-C motif chemokine receptor (CXCR)2 can directly prevent infiltration of MDSCs into the TME and mediate infiltration of T cells. Thus, the combined use of CXCR2 and PD-1 inhibitors significantly prolongs OS in a mouse model of PDAC [108]. Apolipoprotein A-I mimetic peptide L-4F can inhibit the differentiation and activation of MDSCs by downregulating the signal transducer and activator of transcription (STAT)3 signaling pathway of MDSCs. L-4F has the potential to be used as an adjunctive drug for ICI treatment [109]. In 2016, Huang et al. [110] found that ltp-1, another inhibitor of the STAT3 signaling pathway, can inhibit the growth of pancreatic cancer in vivo and in vitro. It will be interesting to establish whether ltp-1 can enhance ICI therapy. In addition to the STAT3 signaling pathway, MDSCs are also regulated by CD200 and colony stimulating factor 1 receptor (CSF1R). Antagonists of CD200 and CSF1R can reduce the proliferation and activation of MDSCs and inhibit the growth of a PDAC model in vivo by combination with ICIs [111, 112]. Another study showed that the combination of CSF1R inhibitors and CXCR2 blockers significantly inhibited proliferation and activation of TAMs and MDSCs and enhanced the therapeutic effect of ICIs on solid tumors [113]. In addition to targeting CSF1R, disruption of the galectin-9/dectin 1 axis can also reverse the immunosuppressive TME caused by M2-TAMs. Zhou et al*.* found that exosomes based on bone marrow mesenchymal stem cells can significantly enhance the efficacy of targeted therapy and downregulate the number of M2-TAMs and Tregs in the TME. In the future, the combination of this new biological therapy and ICIs is worth pursuing [114]. For the other two immunosuppressive components, Tregs have a higher expression of C–C chemokine receptor (CCR)4. CCR4 antibody can induce apoptosis of Tregs. However, the combination of CCR4 antibody and ICIs durvalumab or tremelimumab did not improve the prognosis of patients with advanced solid tumors in a phase II clinical trial. The reason for this is not known and may be related to drug dose [115]. Growth of TAMs/cancer-associated fibroblasts (CAFs) can be directly inhibited by blocking the PAK1 [116] pathway or using xl888 (a heat shock protein 90 inhibitor) [117]. Thus, these two novel therapeutic methods targeting PSCs not only improve T-cell proliferation and infiltration, but also significantly improve the efficacy of ICIs as adjuvants. The small molecule glutamine analog 6-diazo-5-oxo-L-norleucine enhances infiltration of CD8^+^ T cells through downregulation of dense extracellular matrix, which has been proved to have a synergistic effect with PD-1 receptor blockers [118]. By targeting the small molecules secreted by these cellular components, the efficacy of ICIs can also be improved. For example, CXC chemokine ligand (CXCL)12, secreted by CAFs, can induce tumor-cell evasion of immune surveillance by inhibiting the activation of T cells. By inhibiting CXCR4, some authors have discovered that the activity of CD3^+^ T cells can be restored in a synergistic manner with anti-PD-1 drugs in vitro or in vivo [119, 120]. Another study designed to inhibit galectin-1 secreted by PSCs improved the ability of CD4^+^and CD8^+^ T cells to infiltrate the TME. The authors speculated that the infiltration of functional T cells into the TME is the key factor in ensuring the efficacy of immunotherapy [121]. By reconstituting the TME of PDAC, ICIs can more easily eliminate immunosuppression, which also provides a new possibility for combination therapy with ICIs in the future.

### Combination of ICIs and traditional chemoradiotherapy

Chemotherapy with FOLFIRINOX (fluorouracil, leucovorin, irinotecan, and oxaliplatin) and gemcitabine combined with nanoparticle albumin-bound paclitaxel (nab-paclitaxel) remained the first-line treatment for PDAC in 2020 [122]. These drugs exert antitumor effects mainly by affecting the process of tumor cell replication and proliferation. In addition to the traditional cytotoxic effects, they can enhance the therapeutic effect of ICIs on PDAC by enhancing the antigenicity of tumor cells and targeting some inhibitory components in the TME [123]. For example, gemcitabine can downregulate the proportion of MDSCs, Tregs, and TGF-β in the TME of PDAC and increase the number of effector T cells infiltrating the TME [124]. 5-Fluorouracil can reduce the number of activated MDSCs, improve the ability of effector T cells to produce IFN-γ, and promote the efficacy of immunotherapy [125].

The efficacy of the combination of chemotherapy and ICIs has also been confirmed in vivo and in vitro. In a preclinical model of PDAC, the combination of gemcitabine and anti-PD-L1 induced a complete response [126]. Moreover, this combination therapy has been proved to enhance the immune response by increasing the proportion of M1 macrophages and effector T cells in a murine model of liver metastasis [127]. Besides in vitro experiments, some clinical trials have also confirmed that the combination of chemotherapy and ICIs can improve the prognosis of patients with PDAC. In a recent study, Ma et al. [128] found that patients treated with chemotherapy and ICIs had higher OS and PFS than those treated with chemotherapy alone. A phase Ib clinical trial (NCT01473940) also proved that the combination of ipilimumab and gemcitabine could achieve a better prognosis in PDAC patients [129]. Albumin paclitaxel can further improve the prognosis of PDAC patients. In a phase Ib/II clinical trial (NCT02331251), 17 patients who received gemcitabine, albumin paclitaxel, and PD-1 receptor blocker pembrolizumab had an average PFS of 9.1 mo and an average OS of 15 mo [130].

Similar to chemotherapy, PDAC cells are also resistant to radiotherapy owing to the barrier formed by dense matrix. However, the combination of radiotherapy and ICIs can still improve the prognosis of patients, which may be due to the following reasons. First, the tumor antigens on the cells can be exposed by the radiation-mediated tumor cell killing, which is presented by MHC class I and recognized and eliminated by cytotoxic T cells [131]. Second, Valkenburg et al*.* recently found that radiotherapy can reconstruct the matrix stromal components in the TME. Therefore, the immunosuppressive TME of PDAC is changed, which is more conducive to the efficacy of ICIs [132]. This theory is also be supported by the results of some clinical trials. Azad et al*.* used (12, 5 × 3,20 Gy) high-dose radiotherapy combined with ICIs to treat PDAC. Radiotherapy increased the number of activated T cells and upregulated the ratio of CD8:Tregs [133]. The combination of radiotherapy and IDO inhibitors can reduce T-cell depletion, which has a synergistic role with ICI treatment [134]. Many other preclinical and clinical trials have also proved that through combination with radiotherapy, ICIs are more likely to exert their effect of contact immunosuppression and lead to inhibition of tumor growth in vitro and in vivo [135–138]. Finally, another ongoing phase 2 clinical trial (NCT04361162), which combined nivolumab, ipilimumab, and radiotherapy, was conducted in 30 patients with metastatic, microsatellite stable pancreatic cancer. This study started in March 2020 and is currently in progress.

### Combination therapy of two or three antibodies

The combination of two or three immunosuppressants has been shown to improve the prognosis of patients with pancreatic cancer. Rafeal et al*.* confirmed that PD-1 blocker as a supplement to CTLA-4 blocker might alleviate the immune resistance effect of monotherapy and improve the OS of PC patients [139]. The combination of PD-1 and PD-L1 blockers also has a better curative effect by inducing more effector T cells into the TME and generating memory T cells with the function of preventing tumor recurrence [140, 141]. In four patients who received durvalumab combined with tremelimumab, the average OS was increased to 7.18 mo, significantly higher than the mean OS with monotherapy or existing first-line treatment (NCT02527434). A phase 2 clinical trial from 2019 yielded similar results: the objective response rate was 3.1% for patients receiving combination therapy of durvalumab and tremelimumab, compared with no response for patients receiving monotherapy [142]. Another ongoing phase 2 clinical trial, which combines nivolumab, ipilimumab, and radiotherapy, in 30 patients with metastatic, microsatellite stable pancreatic cancer (NCT04361162), started in March 2020. The combination of novel ICIs with antibodies against PD-1, PD-L1, and CTLA-4 has also achieved some success. As a functional monoclonal antibody with LAG-3, TSR-033 can improve the efficacy of PD-1 monotherapy in patients with pancreatic cancer. The combination of LAG-3 and PD-1 receptor antagonists can also enhance the proliferation and infiltration of effector T cells, reversing the immune resistance of the tumor [143, 144]. Similar to LAG-3, the anti-Tim-3 monoclonal antibody (clone m6903) can block the binding of Tim-3 with its three inhibitory ligands. However, Tim-3 receptor blocker monotherapy had no effect on a mouse model of melanoma. Survival can be improved by administering anti-Tim-3 monoclonal antibody and PD-1 receptor antagonist simultaneously [145, 146]. Chauvin et al. [147] have shown that the combination of TIGIT and PD-1 receptor blockers can increase the antitumor activity of CD8^+^ T cells in patients with advanced melanoma and improve prognosis. Ongoing clinical trials include a phase 1/2clinical study (NCT01928394) of combined nivolumab and ipilimumab in solid tumors, including PDAC. In general, the combination of two or three kinds of ICIs may improve the prognosis of PC patients. Additional table files show more information on clinical trials in detail. (Additional file 3 supplements and summarizes the efficacy and tolerability of ICI treatment in clinical studies with existing results in Additional file 2.)

### The “Achilles’ heel” of ICI drugs

Although in some animal experiments and clinical trials, immune checkpoint inhibitors have shown some ability to reverse PDAC immune resistance. But overall, the OS and PFS of most PDAC patients did not achieve significant improvement from this treatment. In addition to the specific immune resistance of PDAC that has been mentioned above, as a new anti-tumor treatment method in recent years, ICIs drug itself has many limitations. First, its immune-related adverse effects (irAEs) as delayed toxicity towards some specific organs of the human body, and this specifical adverse effect seems irrelevant to dose, which means that a lower dose cannot effectively reduce its adverse effects [148]. Second, in addition to the conventional adverse effects of drugs, hyperprogression, which is described by Lancet magazine as the “Achilles’ heel” of immune checkpoint inhibitor treatment, not only increases the mortality of patients in the early stage of immunotherapy but also becomes an uncertain factor on the road to pursue “precision immunotherapy”. Hyperprogression refers to the phenomenon that the degree of disease progression at a rate that far more exceeds than the normal course of this disease in the early stage of treatment. During this period, the degree of tumor progression (volume, speed) and mortality of patients are greatly improved. It is worth mentioning that the super progress phenomenon is not the “patent” of immune checkpoint inhibitor treatment, but its incidence has been greatly improved compared with other treatment methods such as chemotherapy [149, 150]. Although there are few reports on the hyperprogression of immunotherapy for PDAC, we should pay enough attention to it in future research.

## Discussion

Immunotherapy is changing our traditional concept of cancer treatment, and even has become a first-line therapeutic drug in some solid tumors such as non-small cell lung cancer. By relieving the inhibitory effect on T cells, ICIs drugs are expected to tackle problems that cannot be solved by conventional therapy. However, ICIS monotherapy did not effectively improve the prognosis of PDAC patients, which is thought to be related to the suppressive TME and dense extracellular matrix of pancreatic cancer. In addition, we lack effective biomarkers to monitor drug efficacy and guide our selection of drugs. With the progress of research, multi-drug combination therapy seems to bring a glimmer of dawn for PDAC patients. The combined use of chemotherapy, radiotherapy, other immunotherapies including CAR-T and tumor vaccine with ICIs drugs, has improved the therapeutic efficacy of PDAC. However, as a non-immunogenic tumor, the efficacy of ICIs is still limited by the fact that T cells cannot be effectively activated in the TME of pancreatic cancer. For different clinical patients, their tumor antigenicity may have individual differences. The reasonable classification of this population may help us to find the best combination partner of ICI drugs. Some studies have proposed the concept of “immune score” to evaluate the effectiveness of immunotherapy by combining clinicopathological basis with gene sequencing. Peripheral blood TCR profiling also provides a new possibility for early efficacy prediction of ICI. Considering that the immune response is dynamic and changes over time, we need to establish more effective predictors of ICIS regimen treatment response, which may include TIL, IC molecular expression, and many other emerging biomarkers, so as to more effectively and confidently apply ICI drugs to the clinical treatment of PDAC patients. To sum up, we put forward some future directions for improving the efficacy of ICI drugs on PDAC: (1) Increase the initial activation number of T cells, increase the number of tumor-infiltrating T cells and reduce the depletion of T cells; (2) Find more effective biomarkers that can predict the efficacy in a more precise way; (3) Individualized treatment of PDAC patients and monitoring the efficacy in order to find the best combination of ICI drugs.
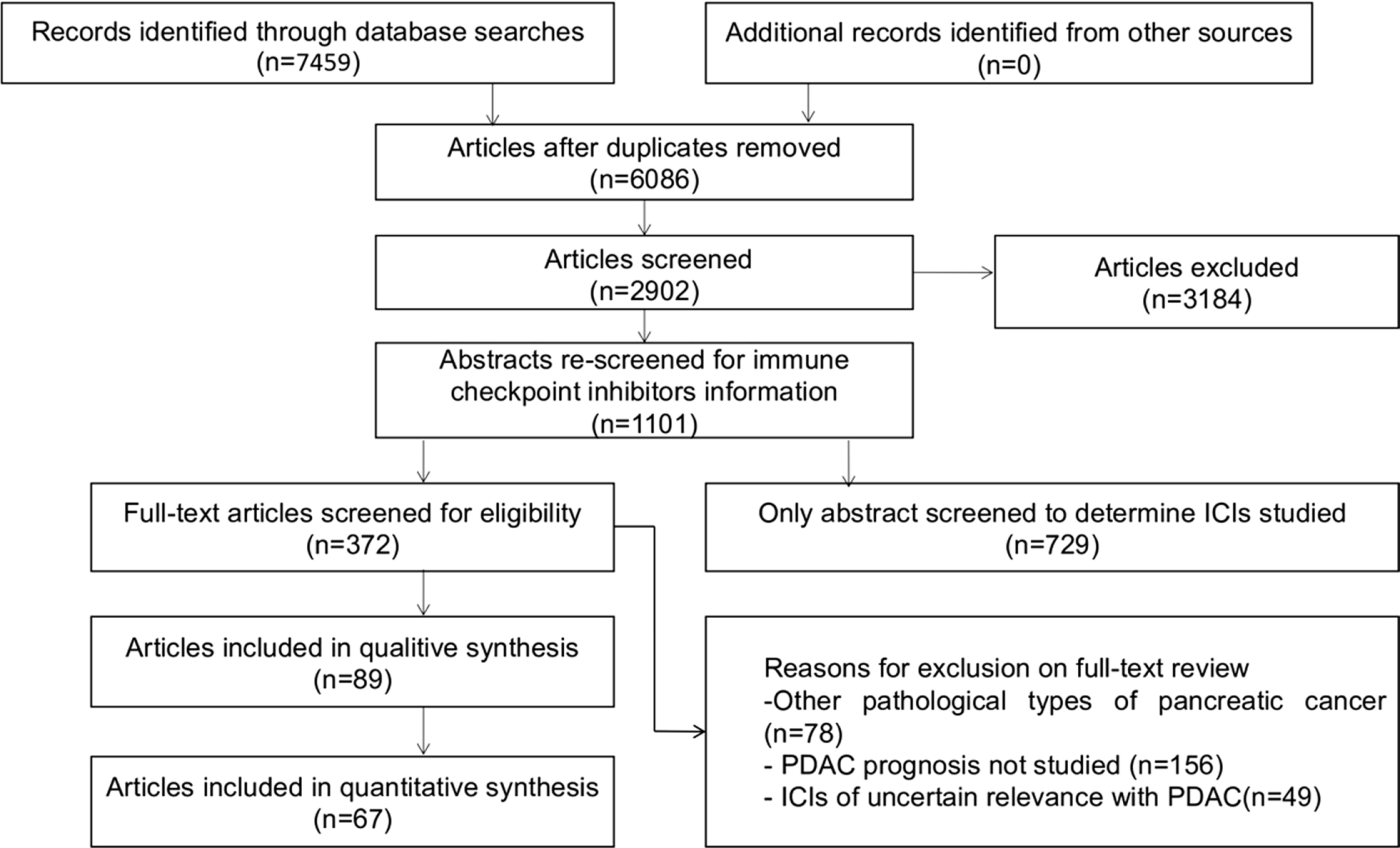


## Supplementary Information

**Additional file 1**. The mechanism of endothelial cell entry into the pancreatic cancer microenvironment and its immunosuppressive effect.

**Additional file 2**. Detailed information of the ICI treatment in the existing clinical studies.

**Additional file 3**. Summary and supplement of the ICI treatment in the existing clinical studies.

**Additional file 4**. Complex tumor microenvironment prevents Tregs from responding in TME when ICIs involved.

## Data Availability

We declared that materials described in the manuscript, including all relevant raw data, will be freely available to any scientist wishing to use them for non-commercial purposes without breaching participant confidentiality.

## Abbreviations

PDAC: Pancreatic ductal adenocarcinoma
ICIs: Immune checkpoint inhibitors
TME: Tumor microenvironment
MDSCs: Myeloid-derived suppressor cells
TAMS: Tumor-associated macrophages
PSCs: Pancreatic satellite cells
OS: Overall survival
PFS: Progression-free survival
PD-1: Programmed cell death protein 1
CTLA-4: Cytotoxic T-lymphocyte-associated protein 4
