# Supplementary figures and images for: Immune checkpoint inhibition for pancreatic ductal adenocarcinoma: limitations and prospects: a systematic review
Source: Cell Commun Signal. 2021 Nov 24;19:117. doi: 10.1186/s12964-021-00789-w (PMC8611916; doi:10.1186/s12964-021-00789-w)

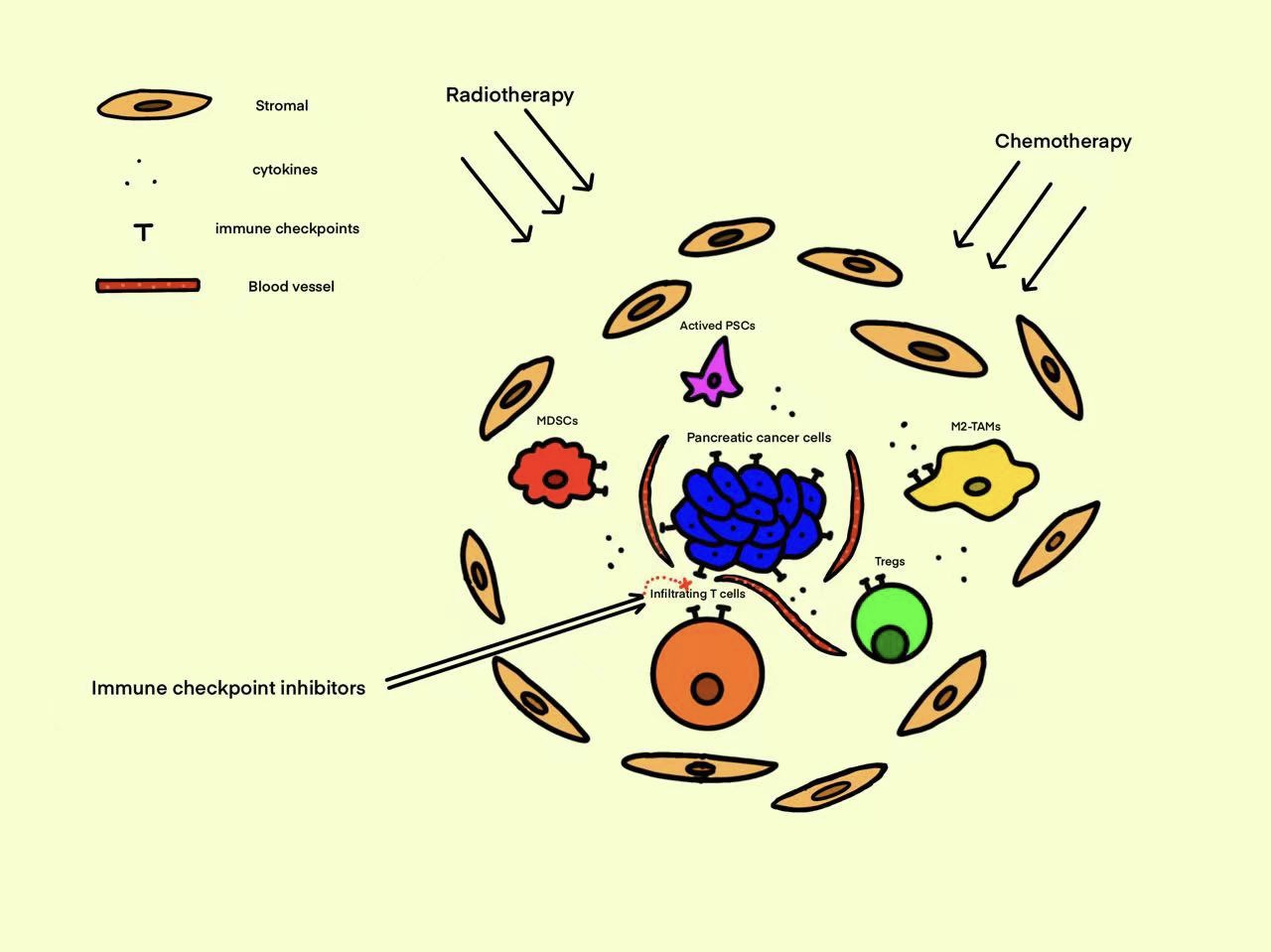

Supplement: Supplementary file 5 — Additional file 4. Complex tumor microenvironment prevents Tregs from responding in TME when ICIs involved. [file 12964_2021_789_MOESM5_ESM.jpg]
